# Large left ventricular thrombus surgically resected in a patient with normal ejection: A case report

**DOI:** 10.1016/j.ijscr.2021.105842

**Published:** 2021-03-24

**Authors:** Abdulkarim Abukhodair, Mohammed S. Alqarni, Abdulmalek Alzahrani, Ziad M. Bukhari, Khalid Zuber, Atif Alzahrani

**Affiliations:** a College of Medicine, King Saud Bin Abdulaziz University for Health Sciences, Jeddah, Saudi Arabia; b King Abdullah International Medical Research Center, Jeddah, Saudi Arabia; c Ghassan Najeeb Pharaon Hospital, Jeddah, Saudi Arabia; d King Abdulaziz Medical City, Ministry of National Guard - Health Affairs, Jeddah, Saudi Arabia

**Keywords:** Left ventricular thrombus, Normal ejection fraction, Tofacitinib

## Abstract

**Introduction:**

A left ventricular thrombus is very rare in a patient with normal systolic function. We are reporting a case of left ventricular thrombus in a patient with an abnormal presentation and normal ejection fraction.

**Presentation of case:**

A 57-year-old female patient presented with severe epigastric and central abdominal pain associated with nausea, vomiting, constipation, and a decrease in appetite. Post-contrast abdominal computerized tomography (CT) scan revealed multiple splenic infarcts. On echocardiographic study, a huge hyperechogenic, mobile mass was seen attached to the septo-apical wall of the left ventricle measuring 20 mm × 40 mm. Magnetic resonance imaging (MRI) also showed transmural late gadolinium enhancement in the apex which is suggestive of small myocardial infarction in the distal left anterior descending territory. Coronary angiogram showed non-occluded coronaries. The patient was treated surgically due to the urgency of the situation and the high risk of embolization.

**Discussion:**

Our patient was on both progesterone and high dose tofacitinib. It is hypothesised that patient most likely experienced asymptomatic myocardial injury with non-occluded coronaries (MINOCA) weeks prior to the presentation. Involvement of distal left anterior descending artery caused apical akinesia resulting in blood stasis. Accompanying subendocardial injury and hypercoagulable state, due to being on progesterone and tofacitinib, led to the formation of the left ventricular thrombus.

**Conclusion:**

Due to the serious outcomes associated with an untreated left ventricular thrombus, it is necessary to carefully assess all patients suffering from systemic embolism with echocardiogram. Assessing the left ventricular mass with MRI was crucial to understanding the nature of the mass. Size, mobility, and protruding nature where characteristics that warranted urgent surgical intervention due to the high risk of embolization. Finally, it is important to note that tofacitinib might be a new suspect for left ventricular clots.

## Highlights

• Left ventricular mass presents with abdominal pain, nausea, and vomiting.
• Left ventricular thrombus can occur in a patient with a normal ejection fraction.
• Tofacitinib flagged last year by the FDA for cardiovascular events.
• Tofacitinib paired with hormonal therapies, old age, & diabetes can cause thrombosis.

## 1 Introduction

A left ventricular thrombus is very rare in a patient with normal systolic function. Some of the factors associated with a left ventricular thrombus include an old myocardial infarction (MI), segmental wall-motion abnormalities, and diseases that increase risk of clot formation [1]. A left ventricular thrombus is usually detected using echocardiography or magnetic resonance imaging (MRI) [2]. Histopathology serves as the modality to identify the source of the mass [2].

The aim of this article is to present a case of left ventricular thrombus in a patient with normal systolic function and to emphasize the challenges of reaching a diagnosis and management plan.

This case report has been reported in line with the SCARE 2020 Criteria [3].

## 2 Presentation of case

A 57-year-old female patient presented with severe epigastric and central abdominal pain associated with nausea, vomiting, constipation, and a decrease in appetite. There was no melena, hematemesis, or respiratory symptoms.

The patient was a known case of diabetes, hyperlipidemia, rheumatoid arthritis, gastritis, inflammatory bowel syndrome, and psychiatric disorder. She had no history of thrombophilia. The patient had been anemic for several months when she presented with vaginal bleeding.

Drug history included Xeljanx (tofacitinib) 11 mg and progesterone for rheumatoid arthritis and vaginal bleeding respectively. She had no history of drug abuse or smoking.

On examination, the patient was stable and afebrile, but she appeared pale and dehydrated. Her pulse was 95, blood pressure was 126/69, and temperature was 37.9. The abdomen was soft and lax with central tenderness. There was no rebound, the chest was clear, and there was no limb edema or signs of deep venous thrombosis. The rest of the physical examination was unremarkable.

For labs, hemoglobin was 6.5 g/dL; reticulocyte count was 2%; amylase and lipase was normal; white blood cells showed lymphopenia; troponin was 0.26 ng/mL; PT was 12.5 s; PTT was 28.6 s.

**Fig. 1:**
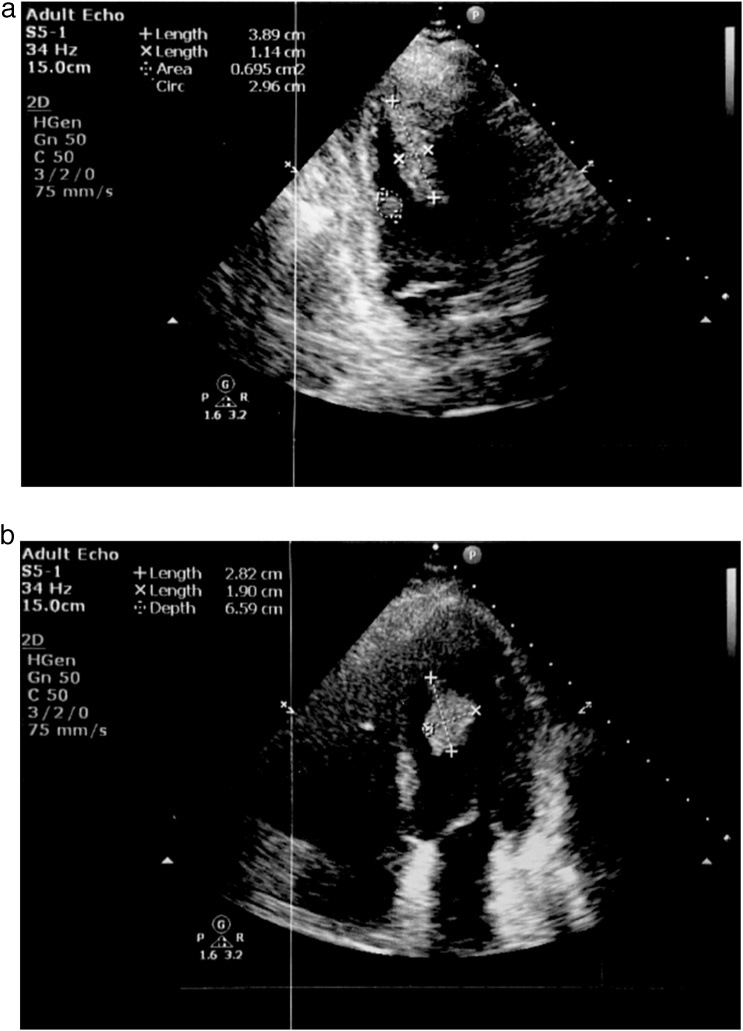
a-b. Echocardiogram of the left ventricle on admission and 10 days after. a Echocardiogram of the left ventricle on admission. b Echocardiogram of the left ventricle 10 days after.

Pre-contrast chest computer tomography showed no abnormalities, but post-contrast abdominal CT revealed multiple splenic infarcts. This increased the suspicion of a left ventricular mass. Doppler echocardiogram showed no obvious segmental wall-motion abnormalities (SWMA), a normal diastolic profile, and a normal ejection fraction (>55%). A huge hyperechogenic, mobile mass was seen attached to the septo-apical wall of the left ventricle measuring 20 mm × 40 mm (Fig. 1a).

The patient was treated for splenic infarction conservatively. She was put on therapeutic anticoagulation for the thrombus which led to vaginal bleeding. After receiving two units of plasma red blood cells and nearly dropping 2.5 gm/dL in hemoglobin since her arrival, she was switched to prophylactic anticoagulation and scheduled for ureteric artery embolization. After an uneventful ureteric artery embolization, the patient was started on unfractionated heparin.

**Fig. 2:**
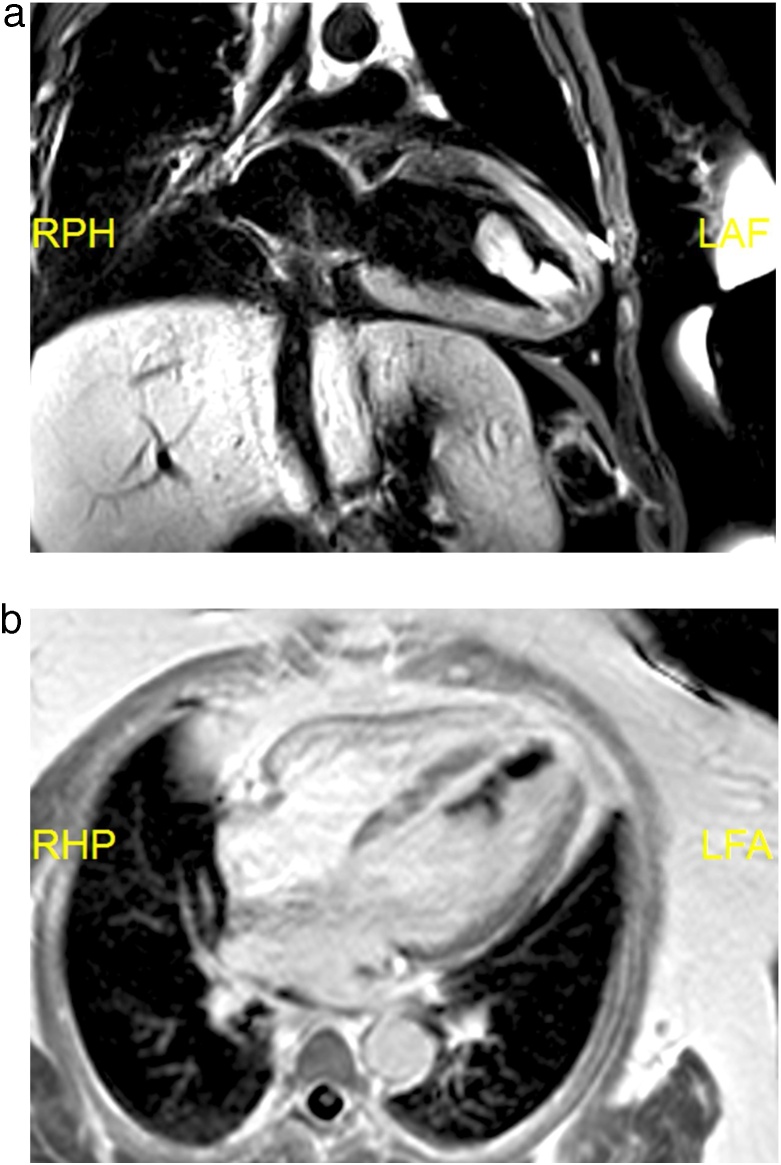
a -b. Cardiac magnetic resonance imaging of left ventricular clot. a Fat-saturated T2 weighted magnetic resonance imaging. b Fixed delayed T1 weighted magnetic resonance imaging at 600 milliseconds showed the mass is dark. This is specific for thrombus.

Cardiac MRI showed a long mobile mass attached to the apex of the left ventricle measuring 15 mm × 50 mm (Fig. 2a-b). The mass did not take contrast on dynamic perfusion sequence, so it was not vascularized. The mass was isointense on T1 images and slightly hyperintense on T2 sequence, but the fix-delayed T1 sequence at 650 ms showed the mass was dark, which is more consistent with a thrombus. The MRI also showed transmural late gadolinium enhancement in the apex which is suggestive of small myocardial infarction in the distal left anterior descending territory.

A 10-day follow-up echocardiogram reported a 14 mm × 33 mm mass with an apical-septal wall attachment of 14 mm in diameter compared to the previous 20 mm (Fig. 1b). Coronary angiogram showed non-occluded coronaries.

Due to the highly mobile and well protruding state of the thrombus, it was flagged with a high risk for embolization. Treatment options were discussed by the medical team and a surgical thrombectomy was decided to be next step.

Preoperative TEE was performed. Surgical approach was through a midline sternotomy. Cannulation was standard ascending aortic right atrial venous cannulation. Antegrade cold blood cardioplegia was used to arrest the heart. Minimum temperature was 32 °C. The non-touch technique of the heart was adopted and cross-clamp was applied as soon as we went on pump.

**Fig. 3:**
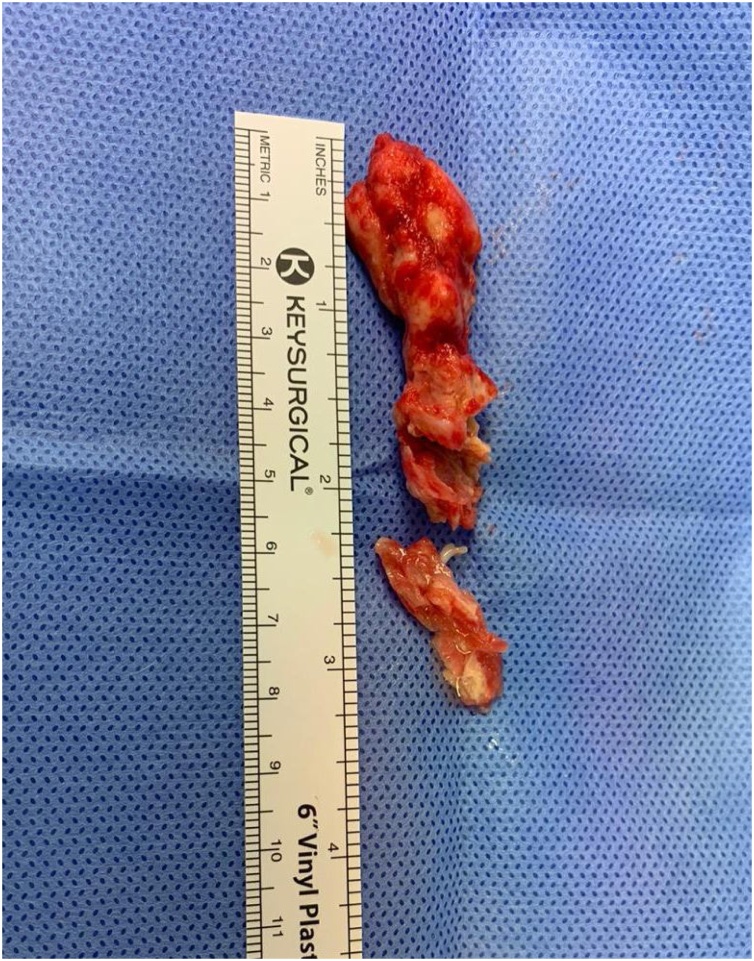
Thrombus in the operating room after being completely resected.

The aortic root was opened transversely, and the left ventricle was examined through the aortic valve by direct vision and using a 5 mm scope to see the clot location and frailty. The mass looked whitish, very fragile and was 60 mm long with a narrow base attached to the left ventricular apex. At this point, we left the aortic root open to make sure we catch any debris coming out from the ventricle. The left ventricular apex was incised in a longitudinal fashion above 1.5 cm lateral to the left anterior descending (LAD) artery. The muscle was opened in layer using gentle dissection with a 15-blade knife. A technique of shaving the endocardium with the attached base of the mass was used and non-touch to the mass. In this manner, the mass was taken out in block without any disturbance to this very fragile longitudinal mass (Fig. 3).

We inspected the left ventricular cavity for any debris before closure of the left ventricular apex in two layers using Teflon filt and 3/0 prolene suture. Attention was then directed towards making sure the aortic root was clean from any particles and was closed using a 5/0 prolene suture.

Patient came off-pump easily with no inotropic support and good hemostasis. A repeat echocardiogram was performed prior to discharge and showed no new thrombus formation.

## 3 Discussion

A left ventricular mass is a generally rare disease that can present in many different ways. The most common cause of intracardiac mass is a thrombus. We must differentiate a thrombus from other causes such as tumors and vegetations. If not detected and treated immediately, a left ventricular thrombus can cause life-threatening embolic complications [4]. Echocardiogram, a non-invasive technique, is the primary imaging modality. However, this test can be inconclusive and warrant a cardiac MRI, a more specific and sensitive test [5].

Although very rare, previously healthy patients with normal ejection fractions, like in our case, can develop left ventricular thrombus. Most cases present with systemic embolic symptoms [[6], [7], [8]]. Our patient presented with abdominal pain and gastrointestinal symptoms. In a case report, pain in the hand and difficultly moving was the presenting systemic embolic symptom [6]. Leg claudication and atypical chest pain have been reported in other reports [7,8].

A left ventricular thrombus is a known complication of heart failure and acute MI. Independent risk factors of left ventricular thrombus after acute MI are lower left ventricular ejection fraction (LVEF), extensive anterior myocardial infarction, severe regional wall abnormalities, and left ventricular aneurysm [9]. Other left ventricular thrombus causes include inherited thrombophilia, protein C and S deficiency, antiphospholipid antibody syndrome, and autoimmune disorders such as lupus erythematosus [[9], [10], [11], [12]]. Our patient was not a case of heart failure or acute MI, and she did not present with any specific symptoms. The incidence of a left ventricular thrombus in such a patient is very rare. We assume the cause of the thrombus to be iatrogenic. High doses of progesterone are associated with an increased risk of thrombosis and clot formation [13]. Similarly, tofacitinib (Xeljanz, Xeljanz XR), a Janus kinase (JAK) inhibitor, has been linked with an increased risk of blood clots with a higher dose in rheumatoid arthritis patients [14], especially when combined with hormonal contraceptives [15]. Our patient was on both progesterone and high dose tofacitinib. It is hypothesised that patient most likely experienced asymptomatic myocardial injury with non-occluded coronaries (MINOCA) weeks prior to the presentation. Involvement of distal left anterior descending artery caused apical akinesia resulting in blood stasis. Accompanying subendocardial injury and hypercoagulable state, due to being on progesterone and tofacitinib, led to the formation of the left ventricular thrombus.

Left ventricular thrombus has the potential for thromboembolism which could lead to life-threatening stroke and MI. For this reason, prompt diagnosis and treatment are essential in minimizing the risk of embolization. Treatment options include surgical thrombectomy, direct oral anticoagulation, vitamin-K antagonists, low molecular weight heparin or unfractionated heparin, and thrombolysis [16]. However, due to the lack of a standardized guideline for the therapeutic approach in left ventricular thrombus patients, we based our management on the thrombus's risk of embolization given the high mobility, large size, and protruding nature of the thrombus.

Severe congestive heart failure, diffuse left ventricular dilatation, systolic dysfunction, previous embolization, atrial fibrillation, and advanced patient age can increase the risk of embolization [4]. Protrusion, mobility, and size of the thrombus increase risk of embolization [[17], [18], [19]]. Our patient was at high risk for an embolic event considering the previous embolic event and the large, protruded, and highly mobile thrombus. Based on the follow-up echocardiogram report, re-assessment of the thrombus showed an increased risk of embolization due to the now small base of the thrombus along with the adjacent hyperkinetic walls. Adjacent hyperkinetic walls are a significant factor for embolization [19].

## 4 Conclusion

Left ventricular thrombi can be suspected in patients with normal ejection fraction. It is necessary to carefully assess all patients suffering from systemic embolism with an echocardiogram. Assessing a left ventricular mass with MRI is crucial to understanding the nature of the mass. Size, mobility, and protruding nature were characteristics that warranted urgent surgical intervention due to the high risk of embolization. Finally, it is essential to add that tofacitinib might be a new causative agent for left ventricular clots.

## Declaration of Competing Interest

Not applicable.

## Funding

Not applicable.

## Ethical approval

The ethical approval for the case report has been granted by the King Abdullah International Medical Research Centre with the reference number RJ20/223/J.

## Consent

Informed consent was obtained, and this has been included in the manuscript.

## Author contribution

Atif Zahrani: Consultant of Diagnostic Cardiology who was responsible for the imaging studies.

Abdulkarim Abukhodair: Leader, initiator, and supervisor of the case report.

Khalid Zuber: Intensive care specialist who treated the patient and was essential in the data collection and proper understanding of the case.

Abdulmalek Alzahrani: Essential part of data collection.

Mohammed Alqarni: writing the manuscript.

Ziad Bukhari: writing the manuscript and referencing.

## Registration of research studies

Not Applicable.

## Guarantor

Abdulkarim Walid Abukhodair.

## Provenance and peer review

Not commissioned, externally peer-reviewed.
